# The Impact of Neuroticism and Daydreaming in the Link Between Attachment and Suicide Risk in Emerging Adults

**DOI:** 10.3390/ejihpe15040052

**Published:** 2025-04-03

**Authors:** Danilo Calaresi, Valeria Saladino, Fiorenza Giordano, Valeria Verrastro

**Affiliations:** 1 Department of Health Sciences, Magna Græcia University of Catanzaro, Viale Europa, 88100 Catanzaro, Italy; v.saladino@unicz.it (V.S.); valeriaverrastro@unicz.it (V.V.); 2 Department of Human, Social and Health Sciences, University of Cassino and Southern Lazio, Viale dell’Università, 03043 Cassino, Italy; fiorenza.giordano@unicas.it

**Keywords:** attachment styles, neuroticism, maladaptive daydreaming, suicide risk, emerging adults

## Abstract

Insecure attachment styles (AS) are related to increased emotional distress, leading individuals to employ unhealthy coping mechanisms. These maladaptive strategies are often linked to impaired functioning, feelings of hopelessness, and isolation, all of which are recognized as risk factors for suicide. This study aimed to examine whether neuroticism and maladaptive daydreaming (MD) sequentially mediate the relationship between AS and suicide risk (SR). A total of 1152 emerging adults (50% female) between the ages of 18 and 25 completed self-report questionnaires. The results revealed that secure attachment (SA) did not show any direct relationship with MD, but it was linked to SR through the mediating influence of neuroticism. Dismissing attachment (DA) did not demonstrate any connections with the other variables. The path among preoccupied attachment (PA) and SR was fully mediated by neuroticism and MD, while the path among fearful attachment (FA) and (SR) was partially mediated by these factors. These findings suggest that addressing neuroticism and MD could be crucial in reducing SR, especially among individuals with specific AS. Additionally, the results underscore the importance of personalized approaches, as interventions may need to be tailored to individuals’ specific AS.

## 1. Introduction

Emerging adulthood is a distinct developmental stage characterized by identity exploration, shifting social roles, and increased autonomy (Arnett, 2007). Indeed, during this period, individuals experience heightened emotional instability, fluctuating self-concept, and evolving interpersonal relationships, all of which can contribute to psychological distress (Reifman et al., 2007). Furthermore, the transition into adulthood also involves significant life changes, such as entering higher education, joining the workforce, or navigating new romantic and social dynamics (Arnett, 2007; Reifman et al., 2007). These stressors can be particularly challenging for individuals with insecure attachment styles, as difficulties in emotional regulation, heightened sensitivity to rejection, and reliance on maladaptive coping mechanisms may exacerbate their vulnerability to mental health concerns, including anxiety, depression, and suicidal ideation (Guarnieri et al., 2015). Understanding how attachment patterns influence suicide risk during this critical period, particularly through mechanisms such as neuroticism and maladaptive daydreaming (MD), can thus provide valuable insights for mental health interventions targeting emerging adults.

Attachment is a profound emotional bond and connection developed between people in close relationships, which emerges early in life from interactions and experiences between newborns and their primary caregivers, such as parents or guardians (Allen, 2023; Saladino et al., 2024b). Attachment styles are psychological patterns that describe how people behave emotionally and behaviorally in intimate relationships, particularly throughout early infancy. These patterns are impacted by the quality of care provided during infancy and have a significant influence on future social interactions and overall well-being (Livesley & Larstone, 2020). The attachment theory of Bartholomew and Horowitz made significant contributions by categorizing four major attachment styles: secure, dismissing, preoccupied, and fearful (Bartholomew & Horowitz, 1991). These styles correspond to distinct patterns of cognition, emotion, and behavior observed in intimate relationships (Bartholomew & Horowitz, 1991). The secure attachment (SA) style is regarded as the most adaptable and healthy attachment pattern (Bartholomew & Horowitz, 1991). Individuals with a SA style tend to have a positive self-image and trust others. They are open to emotional closeness, actively seek intimate connections, and are confident in their partners’ responsiveness and availability. Individuals who are securely bonded usually have high self-esteem and effective emotion regulation abilities. Individuals with a dismissing attachment (DA) style, differently, value independence and self-reliance more than emotional connection (Bartholomew & Horowitz, 1991). They frequently minimize the significance of close connections and may fail to articulate or understand their own need for connection. Dismissive individuals tend to prioritize self-sufficiency and may avoid or limit emotional contact. The preoccupied attachment (PA) style is distinguished by a strong need for connection and great concerns about rejection or abandonment (Bartholomew & Horowitz, 1991). Individuals with this style of attachment often have excessive worry and concern about their relationships, leaning significantly on their partners for reassurance and reinforcement. They may feel anxious and struggle to establish emotional boundaries. The fearful attachment (FA) style combines avoidance and anxiety in partnerships (Bartholomew & Horowitz, 1991). Individuals with this trait seek connection while harboring deep-seated anxieties about rejection and intimacy. They may exhibit contradictory actions, alternating between wanting intimacy and distancing themselves as a preventive tactic against potential emotional injury.

Neurotic traits, like attachment styles, are psychological constructs that have received considerable attention due to their effects on people’s interpersonal interactions and mental health. A personality feature known as neuroticism is defined by increased emotional reactivity, a propensity for unpleasant emotions, and difficulties controlling emotions (Barlow et al., 2014; Giordano et al., 2025b; Verrastro et al., 2024a). Specifically, in our study, we refer to neuroticism as one of the five broad personality traits assessed by the Big Five Inventory (BFI), a widely used model for measuring personality dimensions (Fossati et al., 2011). Individuals with high levels of neuroticism often experience frequent negative emotions, such as worry, fear, and sadness, and may struggle with emotional distress (Barlow et al., 2014; Giordano et al., 2025b; Verrastro et al., 2024a). Neuroticism is thus considered a core trait influencing mental health outcomes, including vulnerability to psychological issues and maladaptive coping mechanisms (Fossati et al., 2011). Given its strong associations with emotional reactivity and psychological well-being, neuroticism plays a crucial role in understanding individual differences in stress responses and susceptibility to mental health issues. Numerous studies repeatedly show that neuroticism and attachment patterns are meaningfully related. Early encounters with caregivers may impact attachment styles and neuroticism by forming opinions about oneself and other people (Hagekull & Bohlin, 2003). According to Shaver and Brennan (Shaver & Brennan, 1992), these ideas may thus have an effect on how emotions are controlled, how people interact with one another, and whether neurotic tendencies emerge. People who have an insecure attachment style, which is characterized by dismissal, preoccupation, and fear, tend to be more neurotic than people who have SA, which is a reflection of their difficulties controlling and regulating their emotions (Eggert et al., 2007). The above-cited research has demonstrated the intricate and multidimensional nature of the interaction between attachment styles and neurotic tendencies. Positivity toward oneself and others, ease in close proximity, and proficient emotion control are traits of a SA style. People who have a SA style typically feel comfortable in their relationships, are quick to ask for and offer assistance, and have comparatively lower degrees of neuroticism (Both & Best, 2017; Surcinelli et al., 2010). DA style, differently, is linked to a propensity to distance oneself from emotional intimacy and minimize the importance of relationships. This attachment style implies a tendency toward self-reliance, emotional detachment, and potentially reduced neuroticism (Both & Best, 2017; Surcinelli et al., 2010). A PA style is characterized by a great desire for intimacy, approval, and rejection anxiety. An overactive attachment system in relationships, anxiety, and uncertainty are common experiences for people with this style. Because PA is related to heightened emotional reactivity and difficulties controlling emotions, it has been linked to higher degrees of neuroticism (Both & Best, 2017; Surcinelli et al., 2010). Finally, a FA style causes internal tensions and emotional upheaval because it combines a need for connection with a fear of rejection. Because those with this attachment style may struggle with both their need for connection and their fear of rejection, it is linked to increased degrees of neuroticism (Both & Best, 2017; Surcinelli et al., 2010).

Researchers in psychology are trying to figure out what causes excessive and intense imagining, as these behaviors can negatively impact psychological health and interfere with day-to-day functioning. Thus, their attention has been drawn to the connection between neuroticism and unhelpful daydreaming (Somer, 2002). Those who have strong, vivid, and compulsive dreams that surpass what is often considered fantasy or imagination are said to engage in MD (Bigelsen et al., 2016). Oftentimes, it develops into an obsessive and excessive interest that takes up a lot of time and energy that might be used to interact with everyday life. MD can negatively impact daily life, interpersonal relationships, and overall well-being (Somer, 2002). According to Zhiyan and Singer (Zhiyan & Singer, 1997), individuals with high levels of neuroticism may use excessive daydreaming as a coping technique or construct a fantasy world that momentarily comforts them when emotional pain emerges. Barlow et al. (Barlow et al., 2014) state that people with high neuroticism frequently struggle to regulate and manage their emotions. As a result, emerging adults may resort more frequently to unhealthy coping mechanisms such as excessive daydreaming in order to manage their emotions (Bigelsen et al., 2016).

Knowing potential predictive factors of suicide is another concern of note, and it is important for effectively intervening in this worldwide public health issue. Suicide is a major global public health concern, ranking among the leading causes of death worldwide. According to recent estimates, more than 700,000 people die by suicide each year, with suicide rates varying across regions due to cultural, economic, and psychological factors (World Health Organization, 2021). Emerging adulthood is a particularly vulnerable period for suicide risk, as mental health disorders may manifest or intensify during this stage (Calaresi et al., 2024b; Giordano et al., 2025a; Pereira et al., 2018). Research consistently links suicide risk to various mental health conditions, including depression and anxiety disorders (Gomes et al., 2019; Pérez-Marín et al., 2024; Scardera et al., 2020). Given the complex interplay between personality traits, attachment patterns, and maladaptive coping mechanisms, understanding these psychological contributors to suicide is crucial for developing targeted interventions aimed at reducing suicide risk among emerging adults. When daydreaming turns into a compulsive practice, it can negatively impact a person’s life and cause extreme anxiety (Somer, 2002). MD’s extreme intensity can lead to significant alienation from reality and trouble engaging with people. Individuals who engage in MD often use it as a coping mechanism to escape real-life difficulties, but this reliance on fantasy may lead to increased social isolation and detachment from reality (Bigelsen et al., 2016). Over time, this withdrawal from social interactions and real-world problem-solving may contribute to heightened feelings of hopelessness, loneliness, and despair, which are highly related to MD (Saladino et al., 2024a; Somer et al., 2016) and are well-established risk factors for suicidal ideation (Selby et al., 2007). Thus, MD may not only serve as an escape mechanism but also reinforce emotional distress, further exacerbating the risk of suicide. Somer et al. (Somer et al., 2017) state that persons who rely too much on unhealthy daydreaming as a coping mechanism may find it more difficult to learn more adaptive coping mechanisms, which may result in higher emotional distress or even suicidal thoughts. Furthermore, the correlation that exists between the risk of suicide and MD might potentially be influenced by additional psychological variables. Daydreaming that is not helpful can lead to the emergence of co-occurring mental health disorders, including dissociative symptoms (Calaresi et al., 2024a; Ross et al., 2020), which have been connected to mental health problems and a higher risk of suicide (Calati et al., 2017; Verrastro et al., 2024b). The complex interplay of circumstances created by the combination of MD and these concomitant psychological issues may further increase the probability of suicidal thoughts and behaviors.

Because attachment styles may have an impact on people’s emotional health, psychology has given them a lot of attention. This study aims to examine the potential mediation roles of neuroticism and MD, two traits linked to unfavorable outcomes, in order to identify some fundamental mechanisms by which attachment styles affect the risk of suicide. Understanding these mediation processes holds important practical implications. By identifying the mechanisms by which attachment styles contribute to suicide risk (SR), interventions and therapeutic approaches can be informed to enhance individuals’ overall psychological health. Targeting neuroticism and MD may offer opportunities to develop interventions that address underlying concerns, promote healthier attachment styles, and reduce the risk of suicide.

**Figure 1:**
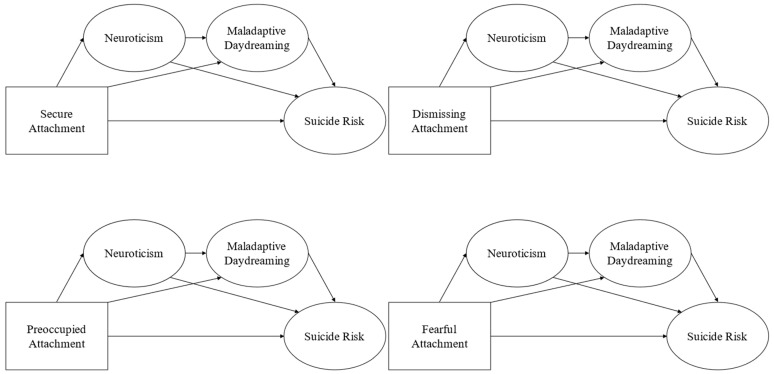
Hypothesized Models.

Given these rationales, this study aimed to examine whether neuroticism and MD sequentially mediate the relationship between attachment styles and SR (Figure 1).

## 2. Materials and Methods

### 2.1. Participants and Procedures

The current study included a sample of 1152 young adults in Italy, with an equal distribution of women (576) and men (576). Participants’ ages ranged from 18 to 25 years (M = 21.48, SD = 2.31). The sample was recruited from several Italian cities. Specifically, a team of twelve trained psychologists, randomly selected and situated across Italy, recruited the participants using both offline and online methods to ensure a relatively high degree of sample representation. A total of approximately 20 psychologists were initially contacted through professional networks and research collaborations. Of these, 15 expressed interest in participating, and 12 were ultimately selected based on their geographical location, professional experience, and availability to ensure a diverse and representative recruitment process. The selection aimed to cover different regions of Italy to maximize sample diversity and avoid recruitment biases. The number 12 was chosen as an optimal balance between broad geographic coverage and logistical feasibility, allowing for effective coordination while maintaining a manageable workload for each psychologist. The age distribution of the sample is as follows: 160 participants (13.9%) were 18 years old, 135 participants (11.7%) were 19 years old, 131 participants (11.4%) were 20 years old, 139 participants (12.1%) were 21 years old, 163 participants (14.1%) were 22 years old, 141 participants (12.2%) were 23 years old, 139 participants (12.1%) were 24 years old, and 144 participants (12.5%) were 25 years old. This distribution reflects a relatively even representation of ages within the emerging adulthood period, ensuring a balanced sample for analyzing psychological factors across different stages of young adulthood. The distribution of relationship status within the sample indicates that the majority of participants were either engaged (43.7%) or single (40.5%), together accounting for 84.2% of the total sample. A smaller proportion of participants reported cohabiting (10.1%), while the least represented group was those who were married (5.7%). Regarding educational background, 17% of the participants completed middle school, 48% high school, 31% university, and 4% postgraduation. In terms of occupation, 45% of the participants were students, 22% were unemployed, 24% were employed, and 9% were self-employed.

Participants were asked to complete an online survey. Participants provided informed consent and participated voluntarily, with no financial incentive. At every stage of the study, participant privacy and confidentiality were given top priority.

### 2.2. Measures

Attachment Styles: Attachment styles were examined with the Italian Relationship Questionnaire (RQ; Carli, 1995), which is a self-report instrument consisting of four items that capture specific attachment styles: secure, dismissing, preoccupied, and fearful. Participants rate items on a 7-point scale (not at all like me-very much like me). Elevated scores on each item indicate higher levels of the corresponding attachment style. Since each attachment style was represented by a single item, it was not possible to calculate Cronbach’s alpha for this measure.

Neuroticism: Neuroticism was measured using the Neuroticism subscale of the Italian Big Five Inventory (BFI-N; Fossati et al., 2011). The BFI-N is an 8-item instrument that investigates neurotic personality traits. Answers are rated on a 5-point scale (strongly disagree–strongly agree). Elevated levels of the BFI-N indicate elevated levels of neuroticism. In this study, Cronbach’s alpha was 0.84.

Maladaptive Daydreaming: MD was evaluated with the Italian Maladaptive Daydreaming Scale (MDS-16; Schimmenti et al., 2020), which is a 16-item self-reported measure that investigates MD tendencies. Answers are rated on an 11-point scale (never or none of the time–all of the time or extreme amounts). Elevated levels on the MDS-16 indicate elevated levels of MD. In this study, Cronbach’s alpha was 0.94.

Suicide Risk: SR was examined with the Suicidal Behaviors Questionnaire-Revised (SBQ-R; Osman et al., 2001), which has demonstrated good validity in Italian populations (Falgares et al., 2017). The SBQ-R is a 4-item self-report measure that captures lifetime suicidal ideation and attempts. The average final score of the SBQ-R can range from 0.75 to 4.5. Elevated levels on the SBQ-R indicate elevated levels of SR. In this study, Cronbach’s alpha was 0.93.

### 2.3. Statistical Analyses

Preliminary analyses were carried out with IBM SPSS 27. The primary analyses, however, were performed using the lavaan package in RStudio 2023.09.1 +494.

In order to investigate the impact of gender, a multivariate analysis of covariance (MANCOVA) was employed, treating attachment styles, neuroticism, MD, and SR as dependent variables, while gender and relationship status were treated as fixed factors and age as a covariate.

A Hybrid Structural Equation Modeling (SEM) approach was utilized to conduct the subsequent analyses. In this approach, attachment styles were treated as observable variables, while neuroticism, MD, and SR were treated as latent variables. Four distinct mediation models, one for each attachment style, were examined. These models included attachment styles as predictors, neuroticism as the initial mediator, MD as the subsequent mediator, and SR as the outcome variable (Figure 1). Indirect effects were evaluated using the bootstrap method with 5000 resamples.

## 3. Results

### 3.1. Preliminary Findings

Table 1 shows descriptive and correlational findings. The observed means in this investigation are consistent with those found in the literature by Tatnell et al. (Tatnell et al., 2018), Gegieckaite and Kazlauskas (Gegieckaite & Kazlauskas, 2022), Schimmenti et al. (Schimmenti et al., 2020), and Lew et al. (Lew et al., 2020).

Initial analyses were carried out to verify the impact of gender, relationship status and age on the variables under investigation. A multivariate analysis of covariance (MANCOVA) was employed for this purpose, and the findings indicated multivariate impacts of gender, Wilks’s λ = 0.98, F(7, 1137) = 3.33, *p* = 0.002, ηp^2^ = 0.02. No multivariate effects of relationship status and age were found. Subsequent univariate ANOVAs showed effects of gender on FA, F(1, 1143) = 12.35, *p* < 0.001, ηp^2^ = 0.01, and on neuroticism, F(1, 1153) = 14.54, *p* < 0.001, ηp^2^ = 0.01. In particular, women participants reported higher levels of FA and neuroticism. Considering these findings, gender was included as a control variable in the mediation analyses.

### 3.2. Mediation Models

The SA model fit the data well, χ2(36) = 237.73; *p* < 0.001; CFI = 0.97; RMSEA = 0.07 (90% CI = 0.06–0.08); SRMR = 0.04. All direct and indirect links were significant, except for the direct path between SA and MD, the direct path between SA and SR, and the indirect path between SA and SR through MD.

The DA model fit the data well, χ2(36) = 230.52; *p* < 0.001; CFI = 0.97; RMSEA = 0.07 (90% CI = 0.06–0.08); SRMR = 0.04. Not all direct and indirect paths between DA and all the other variables were significant. All direct and indirect links between neuroticism, MD, and SR were significant.

The PA model fit the data well, χ2(36) = 233.01; *p* < 0.001; CFI = 0.97; RMSEA = 0.07 (90% CI = 0.06–0.08); SRMR = 0.04. All direct and indirect links were significant, except for the direct path between PA and SR.

The FA model fit the data well, χ2(36) = 226.52; *p* < 0.001; CFI = 0.97; RMSEA = 0.07 (90% CI = 0.06–0.08); SRMR = 0.04. All direct and indirect links were significant.

**Figure 2:**
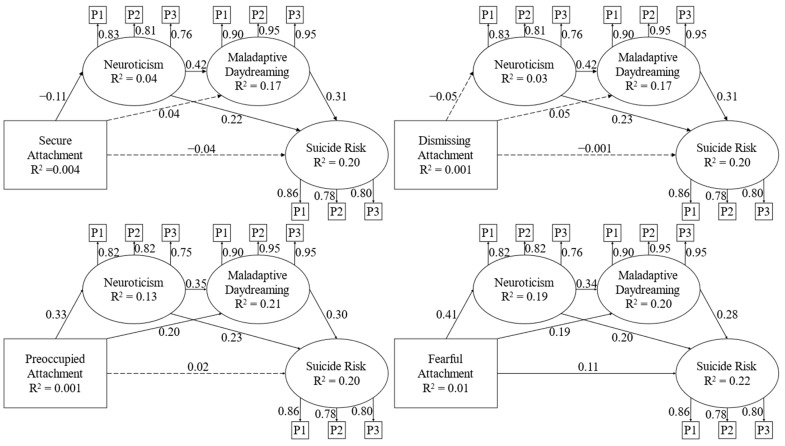
Structural Models. Note: P = parcel; only direct paths are reported for presentation and clarity purposes; paths from gender were not presented for presentation and clarity purposes.

All structural models are shown in Figure 2. All direct and indirect paths of the models are highlighted in Table 2.

## 4. Discussion

This research investigated the mediating effect of neuroticism and MD in the path among attachment styles and SR. The findings of our study unveiled a complex relationship between attachment styles and SR. Specifically, we observed that SA is associated with SR only through the mediating influence of neuroticism. DA, on the other hand, exhibited no direct or indirect relationship with SR. Regarding PA, its connection with SR was fully mediated by both neuroticism and MD. As for FA, the relationship with SR was partially mediated by neuroticism and MD. These findings have important ramifications for our comprehension of the fundamental processes contributing to SR in people with various attachment styles.

The relationship between attachment styles and neuroticism is multifaceted, with distinct patterns emerging for each attachment style. Securely attached individuals, having experienced consistent and supportive caregiving, develop a stable foundation for exploring the world. This security fosters positive internal working models that reinforce interpersonal trust, self-worth, and adaptive coping strategies (Bartholomew & Horowitz, 1991; Livesley & Larstone, 2020). Research indicates that early life experiences play a crucial role in shaping an individual’s psychological development and well-being in later years (Hagekull & Bohlin, 2003; Shaver & Brennan, 1992). These experiences can contribute to the emergence of neurotic tendencies, particularly when attachment security is compromised. Secure attachment fosters the formation of positive internal working models, reinforcing interpersonal trust, self-esteem, and adaptive coping mechanisms. In contrast, individuals with insecure attachment styles may develop distorted or negative internal representations, which can heighten neuroticism, anxiety, and self-doubt (Eggert et al., 2007; Shaver & Brennan, 1992). Additionally, securely attached individuals tend to utilize more adaptive coping strategies, such as effectively managing stress and seeking support when needed, enhancing their overall emotional resilience. However, even among securely attached individuals, variations in emotional reactivity (i.e., neuroticism) may impact self-regulatory abilities. The literature suggests that neurotic tendencies, such as heightened sensitivity to stress or emotional fluctuations, can momentarily challenge SR (Green et al., 2020), explaining why neuroticism acts as a mediator in this context (Eggert et al., 2007). Nevertheless, the overall adaptive coping mechanisms of securely attached individuals may buffer the negative effects of neuroticism on SR, preventing maladaptive coping strategies such as excessive MD (Mariani et al., 2021).

In contrast, DA showed neither a direct nor an indirect association with SR. DA is characterized by emotional detachment, a tendency to downplay attachment needs, and an avoidance of deep emotional engagement (Bartholomew & Horowitz, 1991). The literature suggests that individuals with DA often develop a self-reliant yet emotionally restricted coping style (Craparo et al., 2018), which may limit both the positive and negative influences of neuroticism and MD on SR. Specifically, their avoidance of emotional vulnerability might prevent them from experiencing the heightened distress that typically contributes to neuroticism, thereby reducing the likelihood of engaging in maladaptive coping mechanisms like excessive daydreaming. However, this emotional suppression may also hinder the development of adaptive SR strategies, as DA individuals may struggle to seek support or engage in introspection when needed. This is important to consider due to the significant link between the DA style and interpersonal problems (Stepp et al., 2008), which in turn are highly correlated with suicide-related behaviors (Stepp et al., 2008).

For PA, its link to SR was fully mediated by both neuroticism and MD. PA is characterized by a strong dependence on others for validation, heightened sensitivity to rejection, and difficulties in emotion regulation (Bartholomew & Horowitz, 1991). Research suggests that individuals with PA often experience chronic worry, heightened emotional reactivity, and an excessive focus on relationships, making them particularly prone to neuroticism (Ecer, 2022). This elevated neuroticism can disrupt SR by fostering rumination, emotional instability, and difficulty focusing on long-term goals (Hafferty et al., 2019). Furthermore, PA individuals may resort to MD as a means of escaping emotional distress or compensating for unfulfilled relational needs (Costanzo et al., 2021), reinforcing a maladaptive cycle that impairs SR (Somer et al., 2017). The reliance on MD as an emotional escape further exacerbates difficulties in maintaining attention, delaying gratification, and effectively managing impulses, solidifying the mediating role of both neuroticism and MD in this attachment style (Costanzo et al., 2021; Ecer, 2022).

Meanwhile, FA exhibited a partial mediation effect, with both neuroticism and MD contributing to its relationship with SR. FA individuals experience conflicting desires for closeness and fear of rejection, leading to heightened emotional turmoil and instability (Bartholomew & Horowitz, 1991). Research suggests that this attachment style is strongly associated with neuroticism, as the internal struggle between approach and avoidance tendencies may create chronic anxiety and emotional dysregulation (Troisi et al., 2021). Arguably, these individuals may thus struggle to implement effective SR strategies due to their heightened emotional sensitivity and difficulty trusting their ability to regulate distress. As a result, they may be particularly vulnerable to maladaptive coping mechanisms, including MD, which may serve as a temporary escape from their inner turmoil (Costanzo et al., 2021). Unlike PA individuals, who actively seek external reassurance, FA individuals may oscillate between avoidance and dependence, leading to inconsistent self-regulatory abilities (Bartholomew & Horowitz, 1991), potentially fostering the risk for suicide attempts, as the literature suggests (Macneil et al., 2023). This dynamic may explain why neuroticism and MD partially mediate the relationship between FA and SR and why FA is also directly related to SR.

Research has established a connection between neuroticism and maladaptive daydreaming (MD), as both are linked to emotional regulation difficulties. MD is often employed as a coping mechanism to escape distressing emotions, stressful situations, or real-life challenges (Bigelsen et al., 2016). Individuals with high levels of neuroticism are more susceptible to psychological disorders and frequently experience intense negative emotions (Barlow et al., 2014; Eggert et al., 2007). As a result, they are more likely to engage in MD as a temporary refuge from emotional discomfort. The immersive and vivid nature of MD allows individuals to construct alternate realities in which they feel a sense of control or relief, serving as a psychological escape from real-world distress (Somer, 2002). For highly neurotic individuals, MD can function as an emotional regulation strategy, helping them moderate negative emotions, reduce anxiety, or generate positive imagined experiences (Zhiyan & Singer, 1997). While this may initially provide comfort, excessive reliance on MD can interfere with daily responsibilities and real-world functioning, making it a maladaptive coping strategy (Bigelsen et al., 2016). Neuroticism is also associated with avoidant coping mechanisms, where individuals attempt to evade stressors rather than confront them directly (Barlow et al., 2014). When people use immersive daydreaming as a way to detach from real-life stressors, MD can be viewed as a form of avoidance that hinders emotional growth and effective problem-solving (Alubiady, 2022). This reliance on MD as a primary coping strategy may reinforce neurotic tendencies, creating a cycle of avoidance and emotional distress (Zhiyan & Singer, 1997). Moreover, excessive MD has been linked to heightened suicide risk due to its potential to intensify emotional suffering, disrupt real-world functioning, and contribute to feelings of hopelessness and detachment (Zhiyan & Singer, 1997). While MD may initially serve as a coping tool, its impairing nature can ultimately worsen emotional distress, leading to an increased risk of suicidal ideation (Selby et al., 2007). The disconnection from reality and persistent engagement in fantasy can exacerbate feelings of worthlessness, isolation, and despair, all of which are well-documented risk factors for suicide-related concerns.

The link between suicidal ideation and MD is another one worth noting, especially considering that suicide claims over 700,000 lives annually (World Health Organization, 2021) and that emerging adulthood represents a high-risk period for suicidal behavior (Pereira et al., 2018). When daydreaming becomes compulsive, it can severely affect an individual’s life and trigger intense emotional instability, a heightened intensity which can result in a profound detachment from reality and difficulty connecting with others (Somer, 2002). MD is frequently used as a coping mechanism for intense emotions or difficult life situations, as well as an escape from reality. Hence, it enables people to create elaborate imaginary worlds as a momentary means of escaping their problems (Somer, 2002). However, an over-reliance on daydreaming as a coping mechanism may make it more difficult for individuals to confront and resolve real-world issues (Bigelsen et al., 2016). The act of avoiding problems might potentially heighten emotions of anger and hopelessness, hence raising the risk of suicidal thoughts (Somer et al., 2017). It is also worth underlining that MD can burn time and/or energy, leading to a detachment from real-world events and relationships (Somer, 2002). People may struggle to find meaning or fulfillment in their daily activities when they are unhappy with their current circumstances. As recognized risk factors for suicide thoughts, hopelessness and despair may be exacerbated by this disillusionment and sense of emptiness (Selby et al., 2007). Moreover, excessive and annoying MD may hinder performance in several domains (Bigelsen et al., 2016). Detrimental consequences of diminished functioning may intensify feelings of inadequacy, worthlessness, and frustration, potentially increasing a person’s susceptibility to suicidal thoughts. Lastly, problems with emotional control have been linked to MD (Somer, 2002). Those who dream excessively may do so as a coping strategy to suppress or avoid uncomfortable emotions. Still, there are underlying emotional problems that might worsen emotional discomfort and raise the risk of suicidal thoughts or acts (Somer et al., 2017).

The role of stigma in mental health may also be crucial to understanding the relationship between attachment, neuroticism, maladaptive daydreaming, and suicide risk. Although relationship status and age did not show significant effects in this study, gender emerged as a significant factor influencing the relationships examined. This finding aligns with the growing body of research that highlights how gender norms and societal expectations can influence mental health outcomes. For example, societal stigma surrounding emotional expression and vulnerability often manifests differently across genders, potentially contributing to higher levels of internalized distress in certain individuals (Payne et al., 2008). In our sample, the multivariate effect of gender may suggest that the way individuals engage with their attachment styles and neurotic tendencies may vary between men and women. While stigma related to mental health may be a prominent barrier for both genders, it may be experienced more intensely by women, who may feel societal pressure to suppress their emotional needs or conform to gendered expectations of emotional regulation. On the other hand, men may face stigma related to expressing vulnerability, which could exacerbate emotional difficulties and contribute to maladaptive coping mechanisms (Payne et al., 2008).

### 4.1. Limitations

Our study has several limitations. First, due to its cross-sectional design, we cannot establish causal relationships between the observed associations. Second, the use of self-reported data introduces the possibility of interpretation bias, as participants’ responses may be influenced by their subjective perceptions. Finally, we did not screen for participants’ previous history of mental health disorders, which may have influenced the observed associations.

### 4.2. Future Directions

Future research should address the limitations outlined above by employing longitudinal designs to assess changes over time and incorporating multiple data sources, including clinician reports and objective measurements, to mitigate self-report bias. Additionally, studies should explore the potential effects of different contextual variables, such as social support, which may influence the relationships examined in this study. Investigating these factors could contribute to a more comprehensive understanding of the mechanisms underlying suicide risk.

## 5. Conclusions

The findings of this study provide valuable insights into the complex interplay between attachment styles, neuroticism, MD, and SR. Understanding these associations can inform clinical practice by guiding the development of effective prevention and intervention strategies. From a therapeutic perspective, addressing insecure attachment patterns and promoting adaptive coping and emotion regulation strategies may be beneficial. Mental health practitioners can design interventions targeting neuroticism and maladaptive daydreaming, which may help reduce SR. Moreover, integrating these psychological constructs into screening and assessment tools could improve the early identification of individuals at heightened risk. Public health initiatives may also benefit from greater awareness of these relationships, allowing for the development of educational programs aimed at reducing stigma, promoting help-seeking behaviors, and providing resources for at-risk individuals.

## Figures and Tables

**Table 1:** Descriptive analyses and correlations.

	M	SD	α	1	2	3	4	5	6
1. Secure	3.77	1.74	-	-	-	-	-	-	-
2. Dismissing	3.94	1.83	-	0.19 **	-	-	-	-	-
3. Preoccupied	3.70	1.71	-	0.09 **	0.04	-	-	-	-
4. Fearful	3.93	1.85	-	−0.06 *	0.16 **	0.45 **	-	-	-
5. Neuroticism	3.28	0.80	0.84	−0.12 **	−0.06 *	0.29 **	0.38 **	-	-
6. Maladaptive Daydreaming	3.07	1.98	0.94	0.00	0.03	0.31 **	0.32 **	0.35 **	-
7. Suicide Risk	1.27	0.80	0.83	−0.06 *	0.00	0.16 **	0.26 **	0.26 **	0.36 **

Note: *n* = 1152. * *p* < 0.05. ** *p* < 0.01.

**Table 2:** Path Estimates, SEs and 95% CIs.

	*β*	*p*	*SE*	*CI LL*	*CI UL*
Secure attachment model					
Direct Effect					
Secure Attachment → Neuroticism	−0.11	0.002	0.01	−0.07	−0.02
Secure Attachment → Maladaptive Daydreaming	0.04	0.17	0.03	−0.02	0.11
Secure Attachment → Suicide Risk	−0.04	0.18	0.02	−0.05	0.01
Neuroticism → Maladaptive Daydreaming	0.42	<0.001	0.10	0.88	1.27
Neuroticism → Suicide Risk	0.22	<0.001	0.06	0.16	0.39
Maladaptive Daydreaming → Suicide Risk	0.31	<0.001	0.02	0.11	0.18
Indirect Effect via Neuroticism					
Secure Attachment → Maladaptive Daydreaming	−0.05	0.002	0.02	−0.08	−0.02
Secure Attachment → Suicide Risk	−0.03	0.01	0.01	−0.02	−0.004
Indirect Effect via Maladaptive Daydreaming					
Secure Attachment → Suicide Risk	0.01	0.17	0.01	−0.003	0.02
Neuroticism → Suicide Risk	0.13	<0.001	0.02	0.11	0.21
Dismissing attachment model					
Direct Effect					
Dismissing Attachment → Neuroticism	−0.05	0.10	0.01	−0.05	0.004
Dismissing Attachment → Maladaptive Daydreaming	0.05	0.09	0.03	−0.01	0.11
Dismissing Attachment → Suicide Risk	−0.01	0.82	0.01	−0.03	0.02
Neuroticism → Maladaptive Daydreaming	0.42	<0.001	0.10	0.87	1.26
Neuroticism → Suicide Risk	0.23	<0.001	0.06	0.17	0.39
Maladaptive Daydreaming → Suicide Risk	0.31	<0.001	0.02	0.11	0.18
Indirect Effect via Neuroticism					
Dismissing Attachment → Maladaptive Daydreaming	−0.02	0.11	0.01	−0.05	0.01
Dismissing Attachment → Suicide Risk	−0.01	0.14	0.004	−0.01	0.001
Indirect Effect via Maladaptive Daydreaming					
Dismissing Attachment → Suicide Risk	0.02	0.09	0.004	−0.001	0.02
Neuroticism → Suicide Risk	0.13	<0.001	0.02	0.11	0.20
Preoccupied attachment model					
Direct Effect					
Preoccupied Attachment → Neuroticism	0.33	<0.001	0.01	0.11	0.16
Preoccupied Attachment → Maladaptive Daydreaming	0.20	<0.001	0.03	0.15	0.28
Preoccupied Attachment → Suicide Risk	0.02	0.61	0.02	−0.03	0.04
Neuroticism → Maladaptive Daydreaming	0.35	<0.001	0.10	0.71	1.12
Neuroticism → Suicide Risk	0.23	<0.001	0.06	0.17	0.40
Maladaptive Daydreaming → Suicide Risk	0.30	<0.001	0.02	0.11	0.18
Indirect Effect via Neuroticism					
Preoccupied Attachment → Maladaptive Daydreaming	0.12	<0.001	0.02	0.09	0.16
Preoccupied Attachment → Suicide Risk	0.07	<0.001	0.01	0.02	0.06
Indirect Effect via Maladaptive Daydreaming					
Preoccupied Attachment → Suicide Risk	0.06	<0.001	0.01	0.02	0.04
Neuroticism → Suicide Risk	0.11	<0.001	0.02	0.09	0.17
Fearful attachment model					
Direct Effect					
Fearful Attachment → Neuroticism	0.41	<0.001	0.01	0.13	0.18
Fearful Attachment → Maladaptive Daydreaming	0.19	<0.001	0.03	0.12	0.25
Fearful Attachment → Suicide Risk	0.11	0.001	0.02	0.02	0.08
Neuroticism → Maladaptive Daydreaming	0.34	<0.001	0.11	0.67	1.10
Neuroticism → Suicide Risk	0.20	0.001	0.06	0.13	0.36
Maladaptive Daydreaming → Suicide Risk	0.28	<0.001	0.02	0.10	0.17
Indirect Effect via Neuroticism					
Fearful Attachment → Maladaptive Daydreaming	0.14	<0.001	0.02	0.10	0.18
Fearful Attachment → Suicide Risk	0.08	0.001	0.01	0.02	0.06
Indirect Effect via Maladaptive Daydreaming					
Fearful Attachment → Suicide Risk	0.05	<0.001	0.01	0.02	0.04
Neuroticism → Suicide Risk	0.10	<0.001	0.02	0.08	0.16

Note: *p* level of significance; *SE* standard error; *CI* confidence interval; *LL* lower limit; *UL* upper limit.

## Data Availability

The data presented in this study are available at the request of the corresponding author (the data are not publicly available due to privacy and ethical restrictions).
